# Deep learning-enhanced data-driven gating improves FDG PET/CT clinical image quality

**DOI:** 10.1186/s40658-026-00851-x

**Published:** 2026-04-05

**Authors:** Zoë Wilson, Meghi Dedja, Kuan-Hao Su, Robert Johnsen, Kevin M. Bradley, Daniel R. McGowan

**Affiliations:** 1 Department of Medical Physics and Clinical Engineering, Oxford University Hospitals Foundation Trust, Oxford, UK; 2 GE HealthCare, Chicago, IL USA; 3 Wales Research and Diagnostic PET Imaging Centre, Cardiff University, Cardiff, Wales; 4 Department of Oncology, Oxford University, Oxford, UK

**Keywords:** PET, Deep learning, Gating, Respiratory motion

## Abstract

**Background:**

Respiratory motion can affect PET image quality. One way to reduce motion effects is respiratory gating. The objective of this study, as we seek to further optimise Data-Driven Gating (DDG) algorithms, is to compare two types of DDG phase-gating methodologies: Method-1 has fixed quiescent period offset while Method-2 has an optimised offset for each cycle based on the amplitude of the waveform. The use of Deep Learning is becoming more prevalent for medical images. A previously validated Deep Learning Enhancement (DLE) algorithm will be assessed in conjunction with DDG PET data as an additional method to improve clinical images impacted by respiratory motion. Six reconstructions were assessed: Ungated 3 min (Clinical Standard), Ungated 6 min (Gold Standard), both gating methods with BSREM reconstructions, and both gating methods with OSEM + DLE. These six reconstructions were compared with data from the NEMA IQ phantom, placed on the QUASAR motion platform. Contrast Recovery (CR), Background Variability (BV) and Contrast to Noise Ratio (CNR) were calculated across 6 hot spheres. The same six reconstructions were assessed for 39 FDG PET-CT patient scans with lesions in the lungs or liver. All patients were identified as “high motion” patients with lesions in the region of interest. An experienced radiologist ranked the images and scored them on a 5-point Likert Scale for lesion detectability, diagnostic confidence, and image quality. Lesion maximum standard uptake value (SUVmax) and liver background noise were analysed across all images.

**Results:**

The DLE methods with both gating methods demonstrated significantly better CNR than the ungated images in the phantom data (*p*<0.05). The performance of DLE with DDG data was also supported by the clinician’s preferences and decreased liver noise across the patient reconstructions. Both phantom and patient data indicated a slight preference for Method 2 for patients with irregular breathing.

**Conclusions:**

DLE algorithm effectively produces BSREM-like images from OSEM inputs with data-driven gated data. Clinician preference strongly indicated a preference for the two DDG DLE methods (*p*<0.05). Preliminary results may indicate an improved image quality with cycle-specific phase offset gated images for patients with irregular breathing patterns characterized by breathing period variance.

## Background

Across medical imaging modalities, neural networks have proven highly effective at enhancing image quality, shortening acquisition protocols, and streamlining patient throughput. In whole-body PET/CT, high-sensitivity functional imaging comes at the cost of limited spatial resolution and inherently noisy reconstructions. A previously developed deep learning algorithm (DLE, GE HealthCare) is specifically engineered to smooth faster, noisier reconstructions to match the lesion detectability of advanced penalized-likelihood approaches, without extending reconstruction times, increasing patient dose, or sacrificing diagnostic performance [1].

**Fig. 1:**
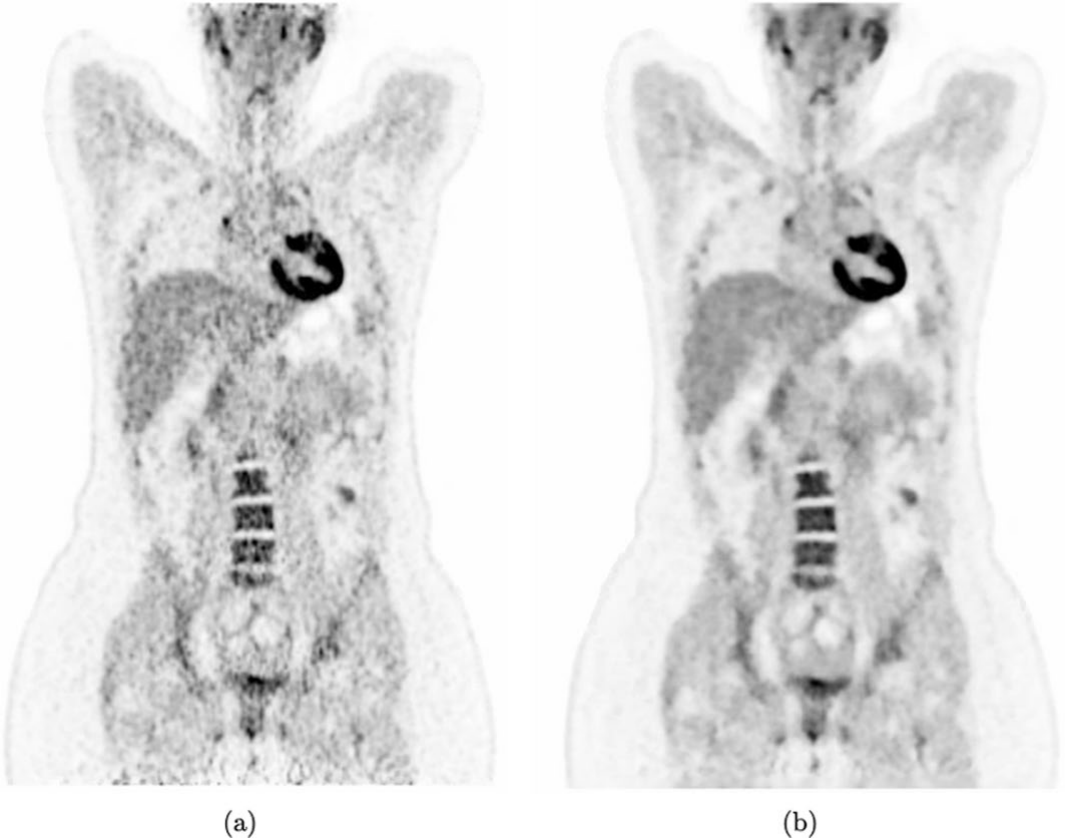
The DLE algorithm was applied to OSEM input image **a**, producing the output **b**

The DLE algorithm is applied to Ordered Subset Expectation Maximization (OSEM) reconstructed images and produces noise reduced images equivalent in lesion detectability and image quality to Block Sequential Regularized Expectation Maximization (BSREM) reconstructed images as seen in Fig. 1. BSREM runs to effective convergence across all beds and so requires a longer reconstruction time (3.2 min/bed position) and higher computational load compared to noisier, faster OSEM reconstructions ( 1.1 min/bed position) [1]. DLE has been previously evaluated on full and partial duration FDG PET images and successfully demonstrated its ability to produce images of clinically diagnostic quality comparable to full counts BSREM images; the current protocol employed at Oxford University Hospitals. Crucially, DLE also mitigates noise in low-count or partial-duration acquisitions - a scenario that frequently arises when applying respiratory motion correction techniques in PET/CT, which inherently discard counts to reduce motion blur.

Respiratory motion can affect PET image quality by causing blurring in the image as well as mismatch between PET and CT images [2]. As a result, in cancer imaging, tumour volumes can be smeared and therefore harder to delineate. This can cause an overestimation of tumour size and underestimation of SUV max, potentially obscuring small tumours within the lungs and upper abdomen [3]. Methods to mitigate the respiratory motion effects acquire a respiratory signal (RS) and use gating mechanisms to synchronize the data with the respiratory cycle [4]. In data-driven gating (DDG), the RS is extracted from the PET list-data itself and has been shown to improve lesion detectability in oncological imaging studies [5, 6]. The usual number of gates is five or six, though some argue that this may degrade image quality as there is a tradeoff between noise incorporation and motion artefact reduction [7].

To compensate for this, Quiescent Period Gating (QPG) was developed, where only 50% of the data is discarded, and the data that is kept is during the ‘quiescent’ part of the breathing which has the smallest degree of motion in the cycle. The inevitable increase in image noise under QPG due to the reduction in the number of counts motivates the integration of DLE with the following two DDG methods. The combined deployment of DDG and DLE for PET reconstruction will be the focus of this study.

Method 1 is a DDG algorithm that uses QPG phase-based gating (Q.Static, GE HealthCare). One limitation of Method 1 is that it uses a fixed phase offset, which has been shown to be less effective than amplitude-based gating for more irregular breathing waveforms [8].

**Fig. 2:**
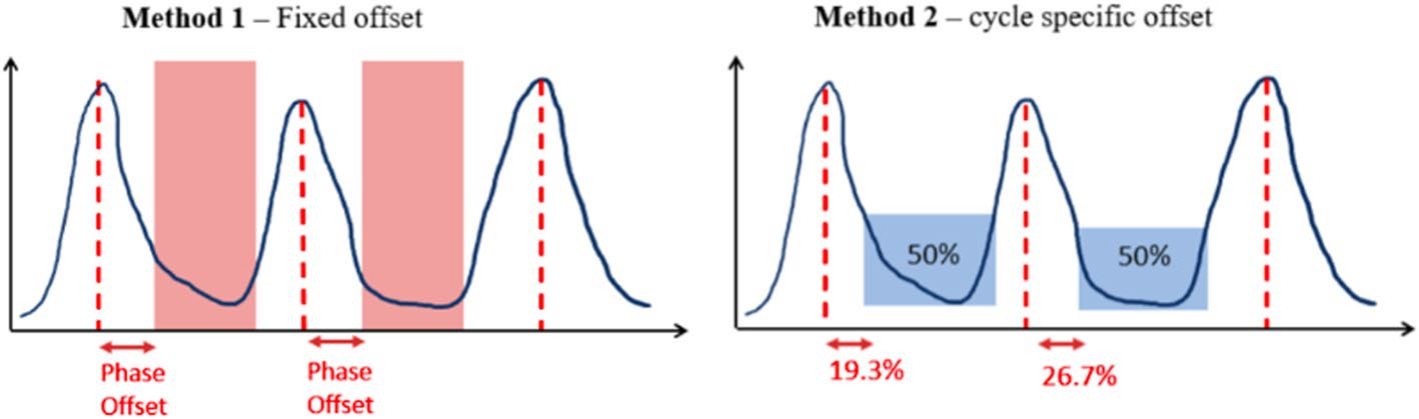
Schematic for Method 1 and 2 on a given waveform with the colour blocks indicating the proportion of the counts identified by the DDG algorithm to be used for image reconstruction

Method 2 is an “optimised offset” gating algorithm based on amplitude-driven phase binning: each gate begins at the cycle’s minimum amplitude and incrementally expands until it encompasses 50% of the total counts, depicted in Fig. 2. We hypothesize that this adaptive amplitude-based gating-combined with DLE noise suppression - will improve lesion detectability and quantification precision in respiratory-gated PET / CT.

## Methods

### Phantom data

The NEMA IQ Body Phantom was filled with F-18 and placed on the QUASAR$^{TM}$ respiratory motion platform (Modus QA: London, ON) and imaged using a Discovery 710 PET/CT scanner (GE HealthCare). The total activity in the NEMA phantom at the beginning of the scans was 123.4 MBq and the contrast between the hot spheres and background was 4:1. The respiratory motion platform was set to be either stationary or moving according to 2 types of respiratory waveforms: one typical type and one irregular type. All waveforms were supplied by the manufacturer and are detailed in Table 1. The phantom was scanned for a 6 min duration and repeated 3 times for each waveform.

For all moving waveforms, the QUASAR hysteresis setting was with no hysteresis. The maximum displacement from the central position was set to be ±15 mm and the direction of movement simulated the motion of abdominal organs during respiratory motion. List data was reconstructed within GE Healthcare’s Duetto offline tools. The data was reconstructed in six ways shown in Table 2. DLE was used outside the bounds of training when applied to phantom images but Reconstructions 5 and 6 are still included for reference.

**Fig. 3:**
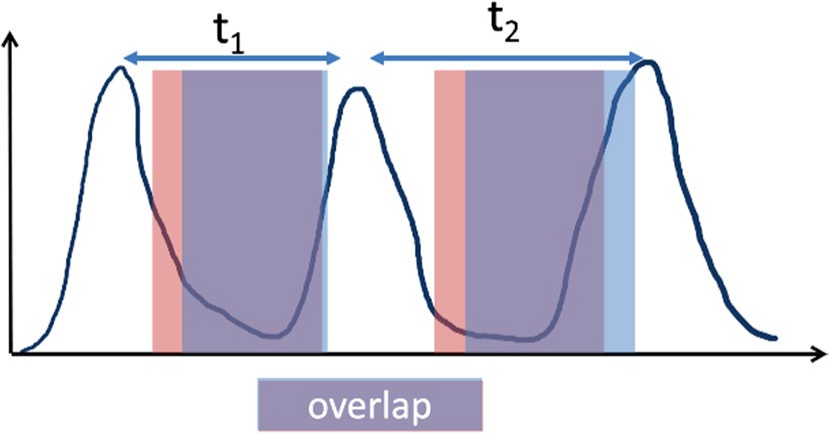
Graphic depicting the regions of overlapping counts gated by two different gating methods shown in red and blue. t1 and t2 show different breathing cycle durations with different overlap values. The standard deviation from the mean period across a full bed position acquisition time (6 min or 3 min) yield the tvar

Duetto was used to calculate the portion of the waveform where both gating methods were applied, referred to as ‘overlap’ and shown in Fig. 3. t var and overlap are extracted from the raw PET list data using Duetto.

The timestamps and sagittal motion amplitudes can be extracted from the tracker data of a given bed position in order to plot the respiratory trace based on the triggers placed by the DDG algorithm. Minimum peak distance of 10 ms and peak prominence threshold (to exclude noise and artifacts) are used to identify the individual breaths, which are then converted into times to compute cycle durations. T variance(tvar) is the standard deviation between all the cycle durations for a scan, producing a quantitative metric indicative of the patient’s breathing regularity. The overlap and tvar for each type of scan is listed in Table 1. When the overlap approaches 1, it signifies that the algorithms used the same portion of the waveform to reconstruct the images, thus resulting in images that are almost identical. A lower overlap value suggests that the breathing is irregular and there is a difference between the portion of waveform gated.

**Table 1:** Summary table for the three different motion types for phantom data acquisitions

Scan No.	Scan type	Waveform type	t-variance ($s^2$)	Overlap fraction
1	Static	no motion	n/a	n/a
2	Regular breathing	Typical9.qrm	1.47	0.86
3	Irregular breathing	Irregular7.qrm	5.06	0.71

Each scan type had three acquisitions, 2 bed positions, and a scan time of 6 min per bed position. The waveform type is listed as it appears in the QUASAR Software

### Patient data

The study used whole-body 18F-FDG PET-CT scans acquired locally between July and August of 2018 on GE’s Discovery 710 or Discovery 690 scanner. Inclusion criteria for the patients included for this study was degree of motion (R>15) and presence of at least one lesion in the lungs or liver. We reviewed 150 cases across these months, and 39 patients fit the criteria (for patient characteristics see Supplementary Materials). The R value is a quantification of the degree of respiratory-like motion which is identified in GE’s DDG algorithms by extracting the signal-to-noise ratio of respiration-like frequencies [9, 10]. A threshold of R>15 is used in GE’s commercially available DDG algorithms to indicate a degree of respiratory-like motion sufficient to warrant motion correction gating and this threshold was applied in this study [11]. The scans with R>15 were selected, with a mean R value for the 39 patients in this study of 20.1 ± 26.6%. The tvar and overlap metrics were also calculated for each of the patient breathing waveforms to indicate breathing regularity.

For each patient, a whole-body helical CT was performed for PET attenuation correction using 100–120 kVp, 150–200mAs. Injected activity was 4 MBq/kg and an FDG uptake time of 90 min.

### Image reconstructions

The BSREM algorithm used was GE HealthCare’s Q.Clear which also includes PSF modeling and used a fixed regularization weighting ($\beta$ = 400) which had been previously optimized [12]. The OSEM algorithm was run with PSF modeling, a standard z filter and no x-y filter, 24 subsets and 2 iterations. All reconstructions were performed with time of flight. BSREM images have been shown to have improved diagnostic capabilities and lower noise compared to OSEM images of the same counts [13]. DLE was applied post-reconstruction to the OSEM images and takes a few seconds to enhance a full body PET/CT.

DLE used 277 F-18 FDG PET and CT images (237 training, 25 validation, 15 testing) collected retrospectively from 6 clinical sites. The algorithm used OSEM reconstructed image inputs, with targets of full duration BSREM reconstructions. The OSEM inputs were reconstructed with a matrix size of 256$\times$256, field-of-view 700 mm, voxel size 2.7$\times$2.7$\times$(2.8 or 3.7) mm$^3$ and 2 iterations, 34 or 24 subsets (depending on scanner type). The regularization factor ($\beta$) for BSREM training data varied depending on clinical protocols of the sites to ensure noise matching across images. Further detail on DLE protocols and model design can be found in Mehranian et al. [1]. These reconstruction parameters were implemented for the reconstructions in this study with $\beta$ value of 400 for BSREM reconstructions and 2 iterations, 24 subsets for OSEM reconstructions.

The 6 reconstructions that were performed on each patient and phantom acquisition are summarized in Table 2.

**Table 2:** Summary of reconstructions whose performance is being investigated

No.	Reconstruction details	Comments
1	Ungated BSREM$_{6\text {min}}$	Extended clinical acquisition–gold standard for study
2	Ungated BSREM$_{3\text {min}}$	Clinical standard
3	DDG Method 1 + BSREM	Commercially available, evaluated previously [10]
4	DDG Method 2 + BSREM	New gating method
5	DDG Method 1 + OSEM + DLE	Computationally fast (60% reduced reconstruction time compared to BSREM).
6	DDG Method 2 + OSEM + DLE	

Both Reconstruction 1 and Reconstruction 2 are ‘ungated’ with no DDG algorithm applied and therefore retain all of the acquired counts from the full duration of their scans. Reconstruction 1 is included as a ’gold standard’ with 6 mins of data for both bed positions (covering the ROI). Reconstruction 2 is the current clinical standard for low motion patients. For high motion patients with R>15, DDG Method 1 protocols trigger the acquisition time per bed position to be doubled for these high R bed positions. After the gating algorithms have been applied to the data collected from these beds, only half the collected counts remain in the quiescent period bins—included in this study as Reconstruction 3. Hence the extension time allows for the same number of counts to be used for the reconstructions across all beds and Reconstruction 1 included as the gold standard for this study.

The doubled acquisition time will 1) provide a reduced noise reconstruction comparison to assess the DLE algorithm’s reduction for half counts equivalent images in Reconstructions 5 and 6, and (2) provide a noise comparison for the two DDG methods. This should facilitate conclusions being drawn regarding whether the additional counts and noise reduction from the ungated extended acquisition in Reconstruction 1 outweighs the benefits of the motion corrected reconstructions with discarded counts in Reconstructions 3, 4, 5, and 6.

DLE and Method 2 are not yet commercially available (Reconstructions 4–6).

### Analysis

#### Phantom analysis

The percentage contrast recovery (CR), background variability (BV), and Contrast to Noise Ratio (CNR) were compared across each sphere across the 6 reconstructions. The ROIs for the hot phantom spheres are drawn as circles of the same inner diameter as the hot sphere. The phantom spheres increase in diameter from 10, 13, 17, 22, 28, and 37 mm. From the background and hot sphere absolute counts values, the contrast recovery (CR), background variability (BV) can be calculated following the equations below with CNR calculated as the ratio CR/BV.

Contrast Recovery in sphere j ($CR_{j}$):

1 $$\begin{aligned} CR_{j} = \frac{\frac{C_{H,j}}{C_{B,j}}-1}{\frac{a_H}{a_B}-1}*100 \end{aligned}$$

$C_{H,j}$ is average number of counts in the ROI for sphere j, is $C_{B,j}$ is average number of background ROI counts for sphere j, $a_H$ and $a_B$ and activity concentration in the hot sphere and background respectively.

Background Variability in sphere j ($BV_{j}$):

2 $$\begin{aligned} BV{j} = \frac{SD_j}{C_{B,j}}*100 \end{aligned}$$

$SD{_j}$ is the standard deviation of the background counts in the ROI for sphere j.

The analysis was conducted across the three repeats and averaged for statistical and visual analysis. To test for significant differences, a Kruskal-Wallis test was carried out followed by Wilcoxon signed-ranks to identify pair-wise comparisons of significance where indicated.

#### Patient data analysis

To assess noise, the standard deviation between voxels and mean number of counts within a 30 mm spherical VOI was calculated using VOI placed in normal, disease-free liver tissue. The same volume was assessed for the mean activity and SD across all 6 reconstructions for each patient, allowing for the signal-to-noise ratio (SNR) to be calculated as in Eq. 3. SUVmax was recorded for any present lesions. Group-wise differences were calculated using the Kruskal-Wallis test. Where indicated, the Wilcoxon signed-ranks test was performed (for ordinal data and SUVmax data) or paired t-tests (for the liver noise) for pair-wise testing with Bonferroni corrections applied to account for multiple comparisons across all reconstructions. All statistical tests were carried out in Python (version 3.11).

3 $$\begin{aligned} SNR = Mean (VOI) / SD (VOI) \end{aligned}$$

An experienced radiologist ranked the 6 reconstructions for each patient. They also scored each image for lesion detectability, image quality, and diagnostic confidence using a 5-point Likert scale [14]. The radiologist’s image assessment focused on small foci ($\le$1 cm) which are more affected by the presence of respiratory motion and would impact diagnosis.

## Results

### Waveforms

For both patient and phantom data, regular and irregular breathing waveforms were characterized and analysed. A lot of different work has addressed the different means of characterizing the degree of respiratory-like motion and breathing regularity [15] [16]. This study focuses on three; R value, tvar, and gating overlap.

**Fig. 4:**
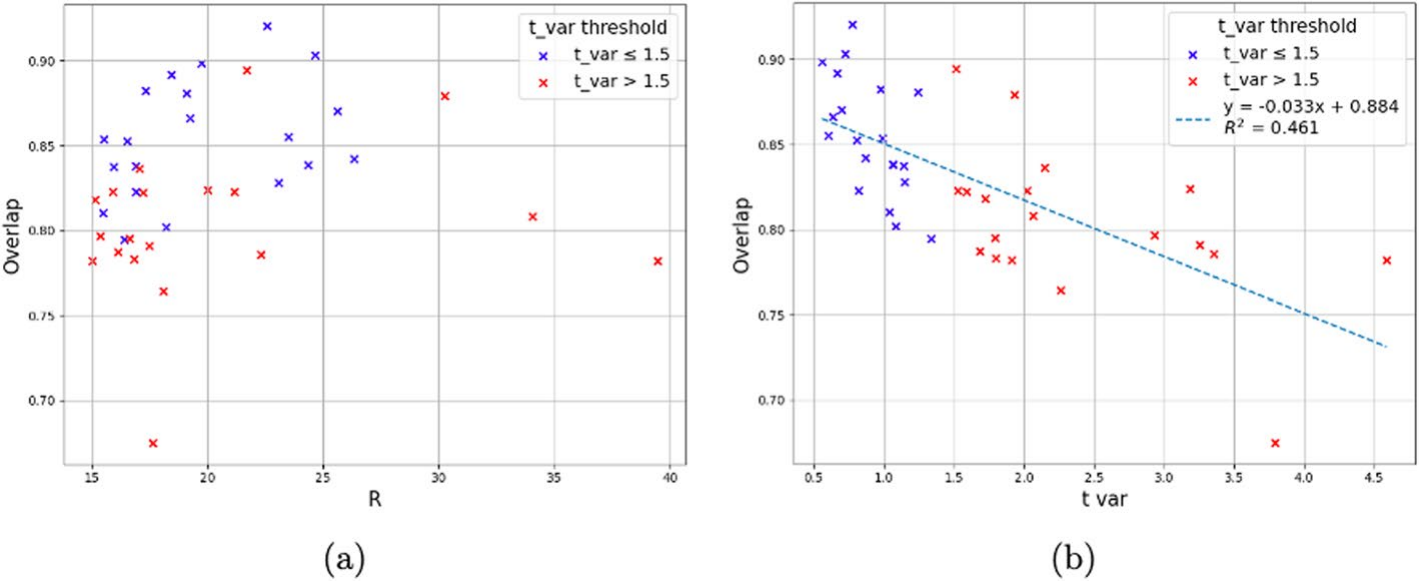
Plots depicting the spread of tvar values for the 39 patients included in this study. **a**: Relationship of R value to overlap and tvar. **b**: Linear regression of relationship between overlap and tvar for this cohort

R value is utilized commercially in Q.Static (Method 1) and DDG methods as a threshold metric to trigger the use of the gating algorithm for a given bed position. Hence R is an indicator of the degree of motion present as opposed to the breathing regularity and has no relationship to tvar or overlap values of the waveforms of the 39 patients in this study, visualized in Fig. 4.

The overlaps of patient waveforms had a range of 67–92%. This is a metric that indicated the proportion of the counts used in both gating methods as illustrated in Fig. 3. A lower overlap percentage indicates a smaller fraction of the counts across a breathing cycle included in the gating by Method 1 and Method 2 and therefore fewer of the same counts utilized in the image reconstruction. A linear regression demonstrated the negative relationship ($R^2$ = 0.461) between the tvar and overlap values. This regression is in line with our assumption that patients with high breathing irregularity (indicated by tvar > 1.5, n=19) would be gated in different ways by Method 1 and Method 2.

### Phantom data

**Fig. 5:**
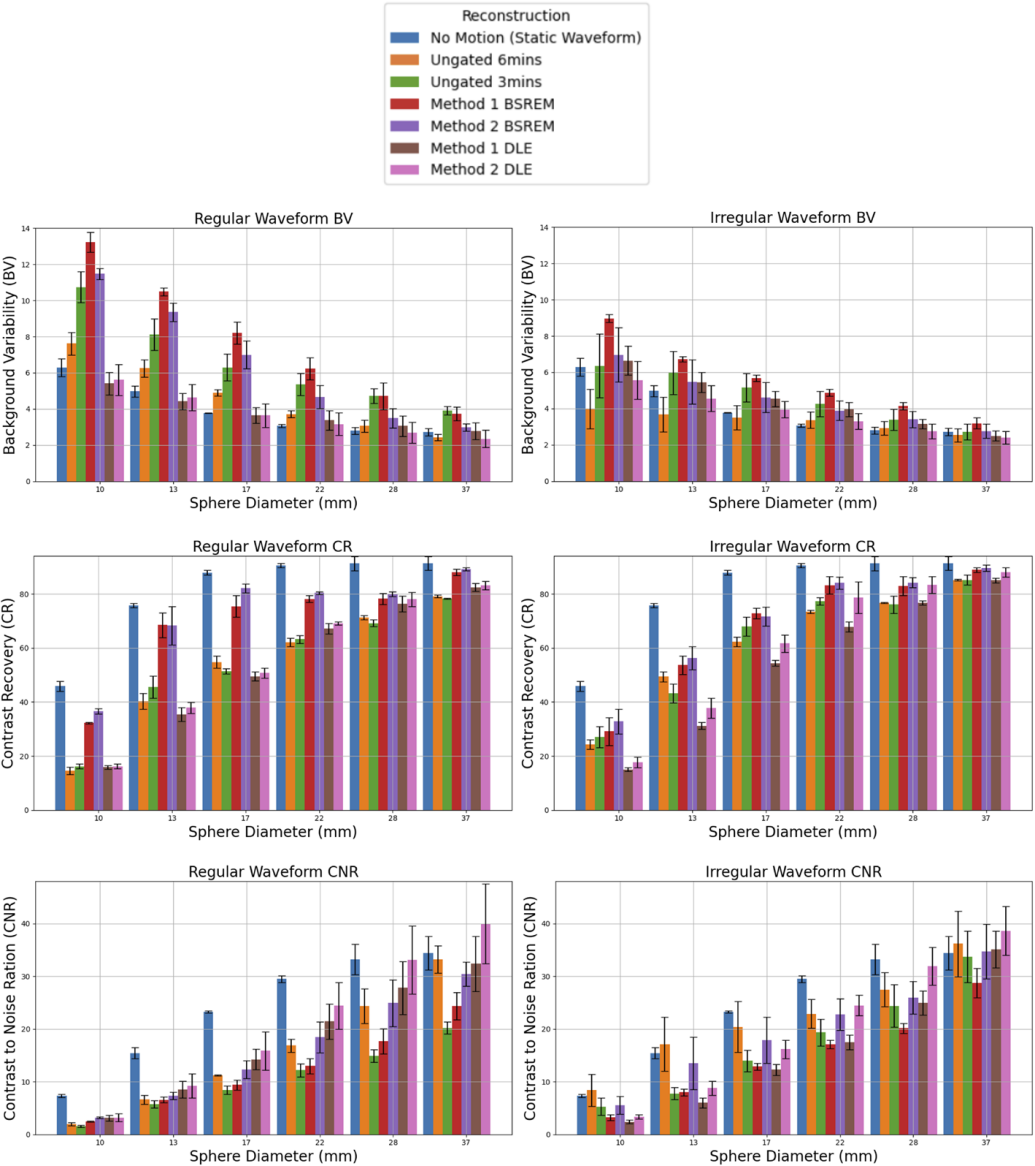
Graphs depicting (from top-down) Background Variability (BV), Contrast Recovery (CR), and Contrast to Noise Ratio (CNR) for the six reconstructions. Left panel: Regular Waveform phantom results, Right panel: Irregular waveform phantom results

The results from the two phantom waveforms are summarized in the mean values of the three repeats per waveform in Fig. 5. The same analysis was also performed on the static full counts (6 min acquisition, shown in blue) phantom as a ground truth for motion corrected data.

Kruskal-Wallis testing indicated a significant difference across the reconstructions for the smaller diameters across CR, BV and CNR. For the irregular breathing waveform, only the 10 mm diameter had significant *p*-value for CNR. CR testing indicated a significance for diameters $\le$ 22 mm and BV $\le$ 17 mm. For the regular breathing waveform, K-W indicated a similarity in CNR and BV across all reconstructions for all sphere diameters, and only a significant difference (*p* $\le$ 0.05) for CR at 13 mm and 17 mm.

The nature of the number of samples per diameter results in all Wilcoxon signed-rank tests being insignificant across these diameters of interest for the three metrics assessed. However, the improved CR for the Method 1 and Method 2 gated images can be seen in Fig. 5 compared to the Ungated reconstructions.

### Patient data

**Fig. 6:**
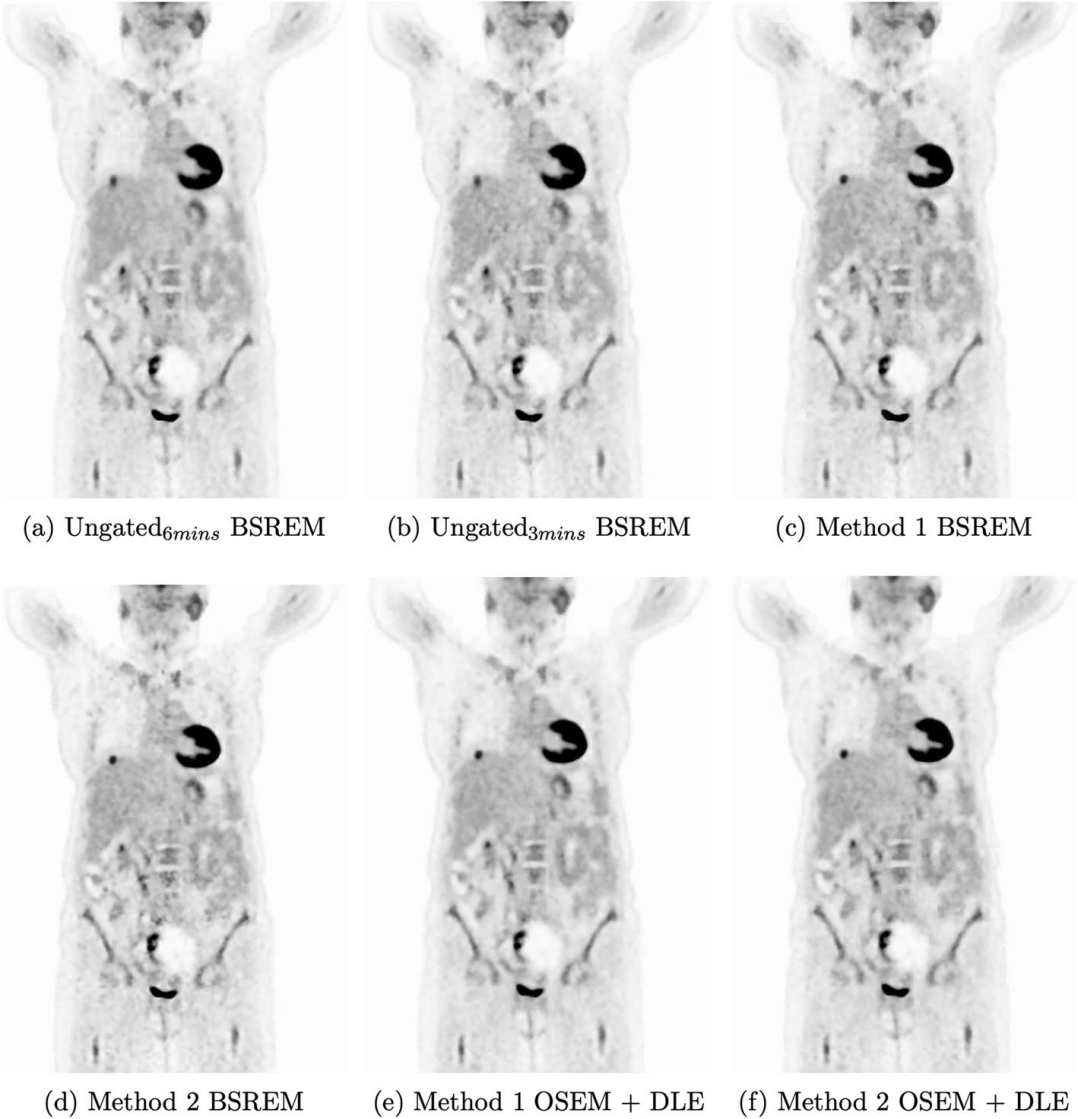
Example series of PET/CT images produced with 6 different reconstruction methods for one of the patients used in this study. These images are PET motion-corrected - CTAC mismatch has not been addressed in this work

Each of the 6 reconstructions for every individual included in this study underwent SUV uptake and liver noise analysis. An example of these six reconstructions is shown in Figure 6. 

#### SUVmax

**Fig. 7:**
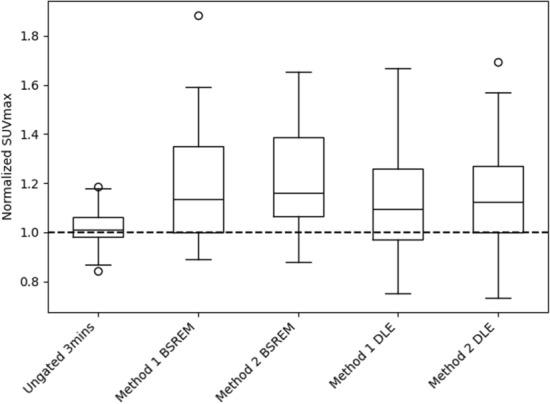
Box plot of mean lesion SUV max scores for 5 reconstructions normalized to the Ungated 6 min image mean SUVmax value

Figure 7 shows the mean SUVmax values for each of the 5 reconstructions, normalized to the gold standard (Ungated 6 min). Statistical testing on the raw (non-normalized) SUV max values demonstrated a non-normal distribution from the Kruskal-Wallis test (*p*<0.05) and so a Wilcoxon signed-rank test was used in the following pairwise comparisons, with the Bonferroni correction for multiple comparisons. Pairwise testing reiterated DDG methods’ higher SUVmax values than half counts ungated reconstructions (*p*<0.05). The only comparable SUV performance in pair-wise comparisons were Method 1 BSREM with Method 1 DLE (*p*$=$0.27) and Method 2 BSREM and Method 2 DLE (*p*$=$0.75). This reiterates the previous work performed with OSEM + DLE demonstrating comparable SUVmax results to the BSREM reconstructions performed within the same DDG method. All other pairwise comparisons showed significant variations across population means.

#### Liver noise

**Fig. 8:**
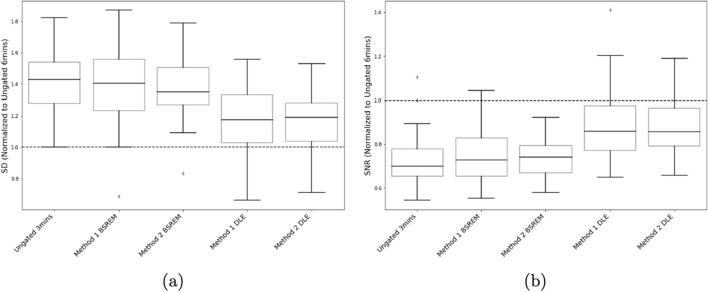
Box plots of mean liver count standard deviation (SD) **(A)** and mean liver SNR **(B)** for 5 reconstructions normalized to the Ungated 6 min image mean value

Both SD and SNR results assessing the liver noise across the 6 reconstructions produced the same improvement in background noise for the DLE image reconstructions. Both Method 1 DLE and Method 2 DLE noise metrics were significantly lower (*p*<0.05) than the ungated 3 min image and both BSREM reconstructions, demonstrating DLE’s improved noise performance compared to the clinical standard. Figure 8 demonstrate the noise metrics for the 5 reconstructions averaged across the 39 patients and normalized to the Ungated 6 min images. The current standard Method 1 BSREM gating method (and Method 2 BSREM) had comparable liver noise levels to the clinical standard 3 min acquisition, as anticipated with the same half counts input to both reconstruction methods. This also demonstrates the efficacy of DLE’s application to the same gated data in comparison.

#### Clinician scores

**Fig. 9:**
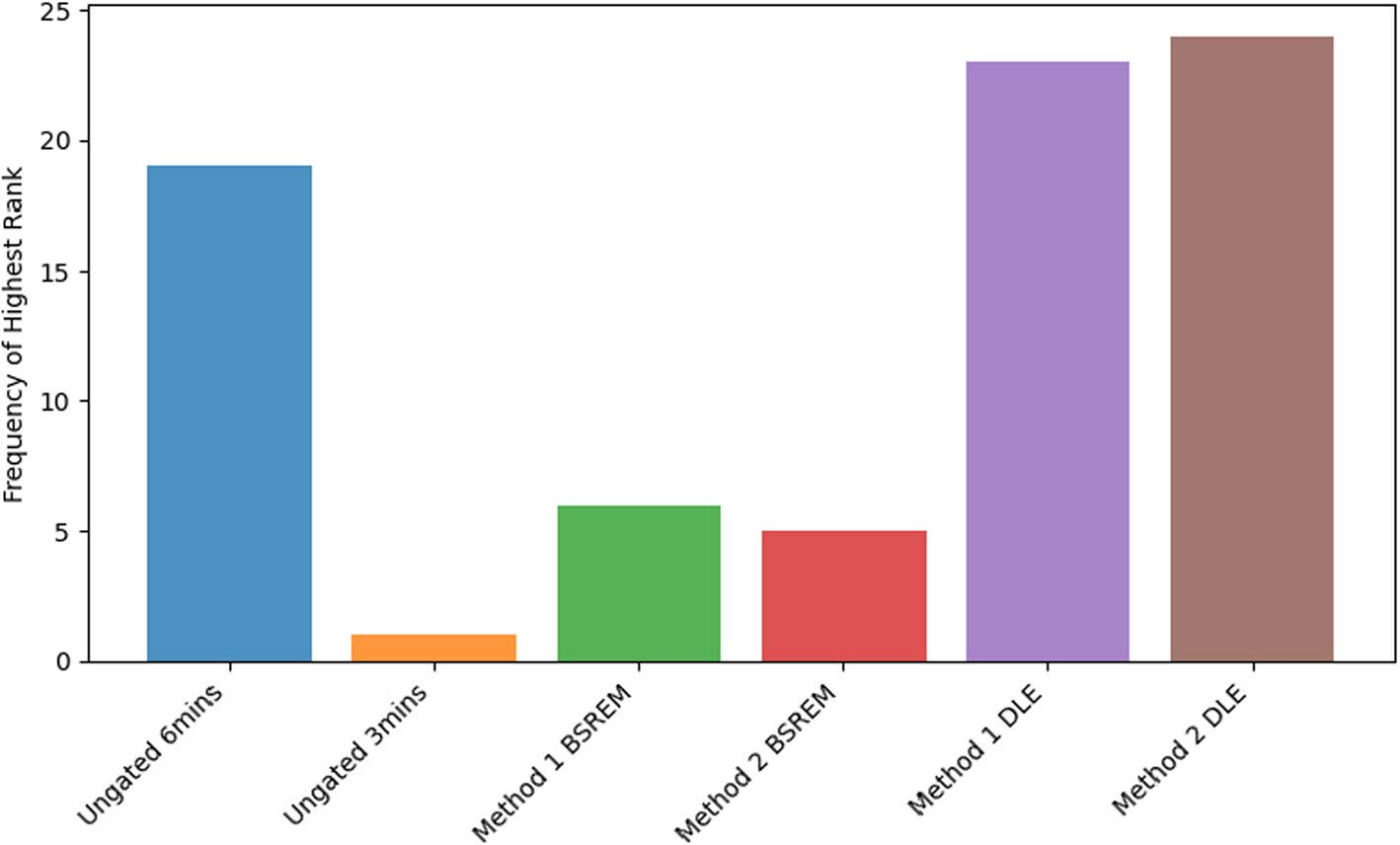
Frequency plot demonstrating the total number of highest rank(=1) awarded to each of the 6 reconstruction methods across the 39 patients

The frequency of highest rank assigned to each reconstruction across the 39 patient image series is shown in Fig. 9. The mean ranks did not accurately reflect the rankings provided by the clinician. The cumulative ‘highest ranks’ awarded exceeds the number of patients in this study as more than one reconstruction was awarded the highest rank in 49% of patients (n=19).

The means and standard deviations of the scores for the full cohort are summarized in Table 3. The highest average score for each metric is shown in bold. This preference for the DLE reconstructions is likewise reflected in the pairwise testing showing a significant increase in qualitative metrics’ scores between DLE-gated and equivalent BSREM-gated reconstructions for both Methods.

**Fig. 10:**
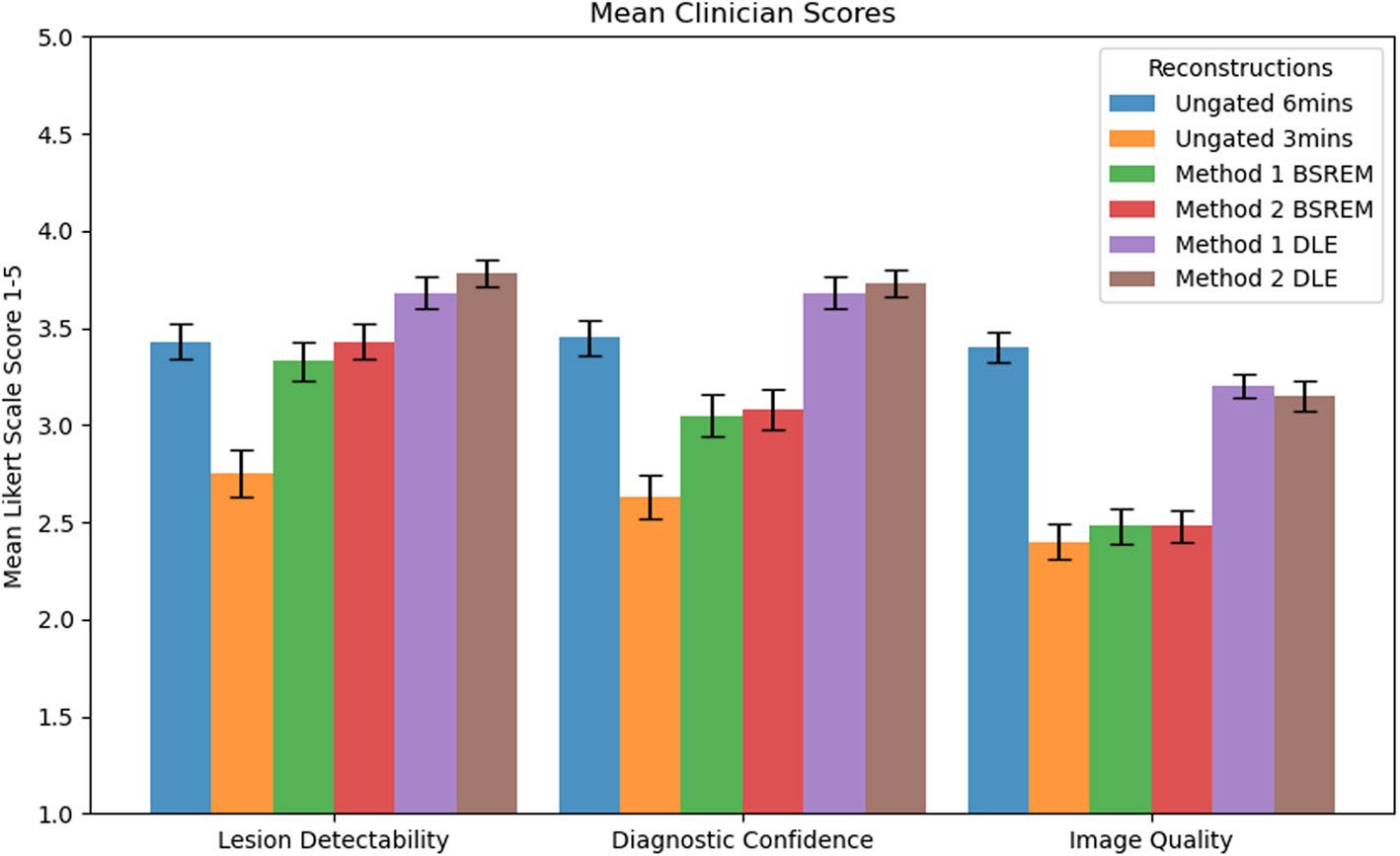
Bar chart depicting the mean and standard deviation of qualitative clinician scores across 6 reconstructions for each of the 39 patients

Figure 10 reflects these mean scores from the clinician for each reconstruction. None of the images were scored as non-diagnostic. Clinician Scores demonstrate a clear preference for the two DLE-gated reconstructions over Static and BSREM reconstructions across all three qualitative metrics and in overall image ranking. The single patient with a surprisingly high rank for the half counts ungated image had excessive motion artefacts that skewed the rankings and were exacerbated on the DLE images.

In Tables 4, 5 and 6, the reconstructions are represented by the following numbers: 1 = Ungated$_{6\text {min}}$, 2 = Ungated$_{3\text {min}}$, 3 = Method 1 BSREM, 4 = Method 2 BSREM, 5 = Method 1 DLE and 6 = Method 2 DLE. Across all three scored qualitative metrics there is no significant difference (*p*>0.05) between the two DDG DLE methods. Likewise, the two DDG BSREM reconstructions demonstrated comparable diagnostic capability and image quality scoring, with borderline lesion detectability. There was also no signficiant difference (*p*>0.05) in Image Quality across the two DDG BSREM methods with the Ungated$_{3\text {min}}$ reconstruction. This highlights the value and efficacy of DLE with DDG Methods in reducing noise in the gated reconstructions, which had significantly higher (*p*$<0.01$) Image Quality Scores than their respective BSREM DDG reconstructions.

**Table 3:** Summary of mean ± SD clinician scores across 39 patients for three qualitative metrics: Lesion detectability, Diagnostic confidence, and Image quality

Reconstruction	Lesion Det.	Diagnostic Conf.	Image Quality
Ungated$_{6\text {min}}$	3.44 ± 0.68	3.46 ± 0.64	**3**.**44** $\boldsymbol{\pm }$ **0**.**55**
Ungated$_{3\text {min}}$	2.85 ± 0.78	2.62 ± 0.71	2.41 ± 0.55
Method 1 BSREM	3.38 ± 0.63	3.10 ± 0.72	2.51 ± 0.60
Method 2 BSREM	3.49 ± 0.60	3.08 ± 0.70	2.49 ± 0.56
Method 1 DLE	3.69 ± 0.52	3.67 ± 0.53	3.18 ± 0.45
Method 2 DLE	**3.77 **$\boldsymbol{\pm }$ **0**.**43**	**3.74 ± 0.44**	3.21 ± 0.52

Reconstructions were scored 1–5 (1 = not diagnostic; 5 = excellent). The highest mean for each metric is emboldened

**Table 4:** Lesion Detectability scores pairwise comparison *p* values table (values rounded to two decimal places)

	1	2	3	4	5	6
1	1	<0.01	**0**.**70**	**0**.**71**	**0**.**08**	0.02
2	<0.01	1	<0.01	<0.01	<0.01	<0.01
3	**0**.**70**	<0.01	1	0.05	<0.01	<0.01
4	**0**.**71**	<0.01	0.05	1	0.05	<0.01
5	**0**.**08**	<0.01	<0.01	0.05	1	**0**.**37**
6	0.02	<0.01	<0.01	<0.01	**0**.**37**	1

Reconstructions are numbered as follows: 1 = Ungated 6 min, 2 = Ungated 3 min, 3 = Method 1 BSREM, 4 = Method 2 BSREM, 5 = Method 1 DLE and 6 = Method 2 DLE. **Bold** indicates *p*$>0.05$

**Table 5:** Diagnostic Capability scores pairwise comparison *p* values table (values rounded to two decimal places)

	1	2	3	4	5	6
1	1	<0.01	0.02	0.02	**0**.**16**	0.03
2	<0.01	1	<0.01	<0.01	<0.01	<0.01
3	0.02	<0.01	1	**0**.**66**	<0.01	<0.01
4	0.02	<0.01	**0**.**66**	1	<0.01	<0.01
5	**0**.**16**	<0.01	<0.01	<0.01	1	**0**.**41**
6	0.03	<0.01	<0.01	<0.01	**0**.**41**	1

Reconstructions are numbered as follows: 1 = Ungated 6 min, 2 = Ungated 3 min, 3 = Method 1 BSREM, 4 = Method 2 BSREM, 5 = Method 1 DLE and 6 = Method 2 DLE. **Bold** indicates *p*$>0.05$

**Table 6:** Image Quality scores pairwise comparison *p* values table (values rounded to two decimal places)

	1	2	3	4	5	6
1	1	<0.01	<0.01	<0.01	<0.01	0.01
2	<0.01	1	**0**.**10**	**0**.**18**	<0.01	<0.01
3	<0.01	**0**.**10**	1	**0**.**56**	<0.01	<0.01
4	<0.01	**0**.**18**	**0**.**56**	1	<0.01	<0.01
5	<0.01	<0.01	<0.01	<0.01	1	**0**.**56**
6	0.01	<0.01	<0.01	<0.01	**0**.**56**	1

Reconstructions are numbered as follows: 1 = Ungated 6 min, 2 = Ungated 3 min, 3 = Method 1 BSREM, 4 = Method 2 BSREM, 5 = Method 1 DLE and 6 = Method 2 DLE. **Bold** indicates *p*$>0.05$

#### Tvar stratification

The clinician scores were then further stratified by an arbitrary tvar threshold of 1.5, with high tvar (>1.5) classified as irregular breathing (shown in Table 7) and low tvar (<1.5) as regular breathing (shown in Table 8) . The lesion scores tables and for regular and irregular breathing cohorts reflect the same shift in preference towards Method 2 for high tvar and Method 1 for low tvar.

**Table 7:** Summary table with mean and standard deviations of clinician scores for patients with more irregular breathing (n=19), indicated by $t_{\textrm{var}}>1.5$

Reconstruction	Lesion Det.	Diagnostic Conf.	Image Quality
Ungated$_{6\text {min}}$	3.44 ± 0.68	3.46 ± 0.64	**3.44 ± 0.55**
Ungated$_{3\text {min}}$	2.85 ± 0.78	2.62 ± 0.71	2.41 ± 0.55
Method 1 BSREM	3.38 ± 0.63	3.10 ± 0.72	2.51 ± 0.60
Method 2 BSREM	3.49 ± 0.60	3.08 ± 0.70	2.49 ± 0.56
Method 1 DLE	3.69 ± 0.52	3.67 ± 0.53	3.18 ± 0.45
Method 2 DLE	**3**.**77** $\boldsymbol{\pm }$ **0**.**43**	**3.74 **$\boldsymbol{\pm }$ **0**.**44**	3.21 ± 0.52

The highest mean values for each metric are emboldened

**Table 8:** Summary table with mean and standard deviations of clinician scores for patients with regular breathing (n=20), indicated by $t_{\textrm{var}}<1.5$

Reconstruction	Lesion Det.	Diagnostic Conf.	Image Quality
Ungated$_{6\text {min}}$	3.40 ± 0.93	3.50 ± 0.89	**3.50 ± 0.85**
Ungated$_{3\text {min}}$	2.80 ± 0.91	2.60 ± 0.82	2.35 ± 0.58
Method 1 BSREM	3.50 ± 0.78	3.05 ± 0.84	2.50 ± 0.71
Method 2 BSREM	3.55 ± 0.84	3.05 ± 0.93	2.50 ± 0.72
Method 1 DLE	**3.80 **$\boldsymbol{\pm }$ **0**.**76**	**3.80 **$\boldsymbol{\pm }$ **0**.**74**	3.20 ± 0.67
Method 2 DLE	3.75 ± 0.44	3.70 ± 0.47	3.20 ± 0.62

The highest mean values for each metric are emboldened

The *p*-values for the respective populations with tvar <1.5 (n=20) and tvar >1.5 (n=19) across the clinician ranks and three qualitative metric scores are shown in Table 9 for the Method 1 DLE and Method 2 DLE reconstruction pair-wise comparisons. Paired difference Shapiro-Wilk tests across these populations indicated a non-normal distribution (*p*<0.05) hence *p*-values in Table 9 are from the Wilcoxon signed-rank test.

**Fig. 11:**
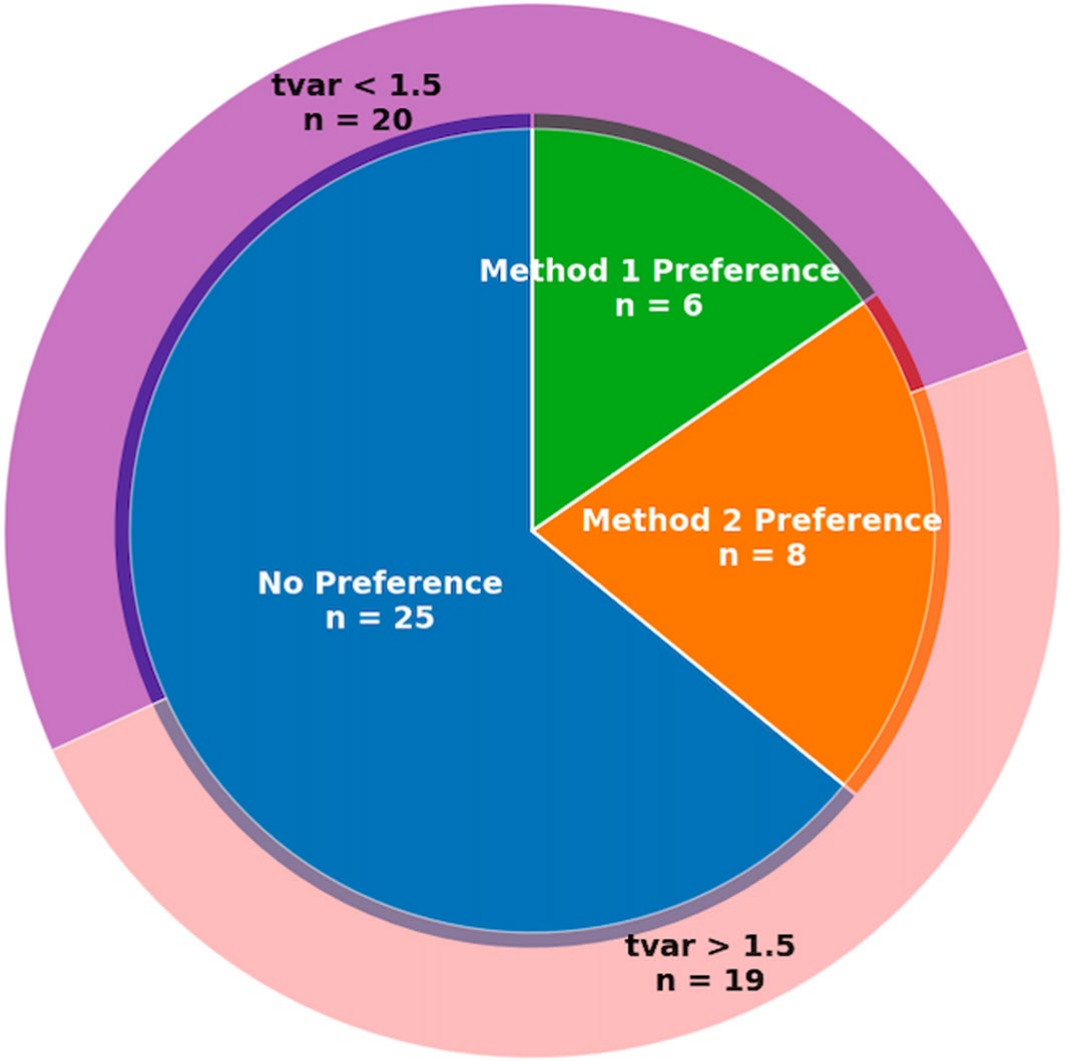
Pie chart indicating the proportion of differential clinician scores for Method 1 DLE or Method 2 DLE and their respective high or low tvar

**Table 9:** Summary table of *p* values from Wilcoxon signed?rank tests between Method 1 DLE and Method 2 DLE for qualitative clinician scores and ranks

Wilcoxon *p* values	tvar	Lesion Det.	Diagnostic Conf.	Image Quality	Ranks
n=20	$<1.5$	0.65	0.41	1.00	0.26
n=19	$>1.5$	0.10	0.06	0.16	**0**.**03**

*p* values below the significance level of 0.05 are shown in bold

Assessing just the two DDG DLE methods, clinician’s rankings of the two methods varied following the pie chart split in Fig. 11. The majority of patients had the same ranking for both gating methods with DLE (n=25). The preferences for either method were counted as any ranking higher than the other i.e., for Method 1 ranked 1 and Method 2 ranked 3, that patient would be marked with a preference for Method 1. Of the 19 patients with tvar>1.5, the clinician preferred the Method 2 DLE image over the Method 1 equivalent in 8 of the cases (32%). A preference for Method 1 DLE was only demonstrated in the low tvar cohort in 6 of the patients (30%). No high tvar patients had a clinician rank that indicated a preference for Method 1 gating with DLE over Method 2.

## Discussion

In this study, the novel application of a Deep Learning Enhancement algorithm was applied to a cohort of 39 FDG PET/CT scans to qualify its performance with data-driven gated data and evaluate the relative performance of two DDG methods. DLE’s aims to reduce noise and produce BSREM-like image quality from OSEM inputs was shown across its application to both the fixed and cycle-specific gating methods.

No previous work has looked at the sequential deployment of these motion compensation and denoising techniques to validate the combined efficacy of their mutual deployment. DLE has not be utilized on respiratory gated data and this study’s assessment of DLE with two DDG methods has shown the algorithm’s utility across these different portions of PET data. The goals of the DLE and DDG algorithms work in parallel. The application of DLE to a reduced counts DDG reconstruction would produce comparable image quality for motion corrected images without the need to extend acquisition times. The investigation into the differential performance of the two gating methods is more informative, when combined with the DLE methodology, previously demonstrated to improve quantitative and qualitative clinical image quality.

SUVmax results affirm the performance of both gating methods with DLE as comparable in quantitative performance to the BSREM equivalent and improved compared to ungated reconstructions. Background noise metrics showed DLE images achieved comparable liver volume of interest SD and SNR to the gold standard (extended, 6 min acquisition) despite having half the photon counts used to reconstruct those images and with OSEM inputs. The preference for the data-driven gated OSEM+DLE reconstructions was demonstrated across both rankings and image scores by an experienced clinician. This agrees with previous work involving this enhancement but has now been demonstrated to successfully both reduce noise and produce BSREM equivalent DDG reconstructions from DDG OSEM inputs [1]. This validation of DLE’s capability in this context is affirming for clinical applications moving forward with both the lower computational load and reduced reconstruction time for patients with respiratory motion undergoing PET/CT scans.

Phantom data assessed visually suggested a consistently higher liver noisier in Method 1 images compared to Method 2, driving down the CNR. However, the variation in quantitative results across the three repeated phantom scans caused minimal statistical significance to support this observation.

The secondary goal of this study, to compare the performance of the two DDG methods, required further cohort stratification to indicate any preference between them. For the total cohort of 39 patients a preference was not evident between DDG images for either Method 1 or Method 2 that was significant across either the qualitative clinician’s scores or image rankings.

Further stratification of the cohort based on overlap (fraction of the patient waveform utilized by both gating methods) similarly did not demonstrate a preference for either method. The variance in the period of patient breathing cycle (tvar) was also extracted from the patient listmode data along with the overlap fraction. An arbitrary tvar threshold of 1.5 $\textrm{s}^2$ was identified: a low tvar under 1.5 (n=20) indicative of a regular breathing pattern and a high tvar greater than 1.5 (n=19) indicative of irregular patient breathing.

Statistical testing comparing the two DDG DLE methods across the two tvar threshold populations likewise yielded only a significant difference (*p* = 0.03) between the ranks of the two gating methods for the high tvar population. The population of more regular breathing patients had no statistically significant differences between Method 1 or Method 2 with DLE across all 3 qualitative clinician scores and the clinician ranks.

The preliminary indications towards a preference in irregular breathing patients for Method 2 with DLE is present in the patient data as well but would require a wider cohort in order to establish this improvement with clinical significance and greater statistical power. These findings support a personalized PET/CT pipeline: extract patient breathing variability (tvar), choose fixed-offset gating for regular patterns or amplitude-based gating for irregular patterns, then apply DLE denoising. This would reduce reconstruction time and avoid additional time in the scanner for patients while achieving gated motion compensated images without any loss of clinical utility. This would also be advantageous as it would allow for more predictable scheduling of studies due to consistent overall study acquisition times.

These challenges mean that patients can experience motion-induced artefacts throughout their clinical journey–from diagnosis and staging to treatment planning and response assessment [3, 17]. By defining a threshold based on breathing variability (tvar), scanners could prospectively flag cases where adaptive, amplitude-driven phase gating (Method 2) is likely to yield superior image quality and this could then go on to inform subsequent imaging. Such a workflow could be embedded in the scanner console, promoting real-time decision support and personalized imaging protocols.

With the 39 patient cohort in a 150-patient sample, 8% (n=12) demonstrated a clinical preference for the DDG DLE method predicted by the tvar threshold, representing 31% of the cohort analysed within our selection criteria. Further prospective studies would also allow for a more realistic quantification of the potential clinical impact for these patients being diagnosed with lung and liver lesions. Whilst already a significant proportion of cases, our selection criteria used ungated images to identify a lesion in the region of interest. With the efficacy of gating and DL enhancements identifying lesions otherwise not visible on ungated reconstructions, it is feasible that a prospective study with these methods would identify disease that would not otherwise have been detected from an ungated FDG PET/CT alone, impacting patient treatment pathways and outcomes [18].

It is possible to have additional motion artefacts as a result of attenuation correction mismatch due to the arbitrary respiratory phase in which the CT is acquired (eg Fig. 6) which could impact the clinician’s scoring and lesion quantification. This would impact all of the six reconstructions being assessed in this study. Previous work has demonstrated the improvement in SUV and lesion quantification when different methods have been applied to both Static and DDG PET images [2, 19–21]. Future work would extend this reconstruction and post-processing motion reduction pipeline to include CT mismatch mitigation methods that have been previously evaluated.

Similarly, a larger dataset would enable a more rigorous determination of the optimal tvar threshold–rather than the arbitrary 1.5 $\textrm{s}^2$ cut off selected here – and would benefit from validation across multiple sites and readers.

## Conclusion

This study validated the performance of a Deep Learning Enhancement algorithm with data-driven gated PET data with both qualitative and quantitative methods. Results comparing the two DDG methods suggested cycle-specific phase offset gating improved lesion detectability and had a clinical preference for irregular breathing patterns but requires further investigation. The ultimate goal of the cycle specific phase offset DDG comparison was to discern an optimized gating and reconstruction pipeline for patients with more irregular breathing patterns.

Our study shows that in combining the use of DLE with DDG data, we achieve motion-corrected PET images in a third of the time of BSREM–without tuning regularization parameters or increasing dose–while maintaining clinically acceptable noise levels. Future multicentre validation and reader studies will refine the tvar threshold and confirm clinical impact for patients with irregular breathing.

## Additional file

Supplementary file 1 (pdf 123 KB)

## Data Availability

The analysed data may be made available by the corresponding author upon reasonable request.

## References

[1] Mehranian A, Wollenweber SD, Walker MD, Bradley KM, Fielding PA, Su K-H, et al. Image enhancement of whole-body oncology [18f]-fdg pet scans using deep neural networks to reduce noise. Eur J Nucl Med Mol Imaging. 2022;49(2):539–49. 34318350 10.1007/s00259-021-05478-x PMC8803788

[2] Cook EL, Su K-H, Higgins GS, Johnsen R, Bouhnik J-P, McGowan DR. Data-driven gating (ddg)-based motion match for improved ctac registration. EJNMMI Phy. 2024;11(1):42. 10.1186/s40658-024-00644-0 PMC11554991 38691232

[3] Noto B, Roll W, Zinken L, Rischen R, Kerschke L, Evers G, et al. Respiratory motion correction in f-18-fdg pet/ct impacts lymph node assessment in lung cancer patients. EJNMMI Res. 2022;12(1):61. 36107357 10.1186/s13550-022-00926-7 PMC9478021

[4] Thielemans K, Rathore S, Engbrant F, Razifar P. Device-less gating for pet/ct using pca. In: 2011 IEEE nuclear science symposium conference record. IEEE; 2011. p. 3904–10.

[5] Dias AH, Schleyer P, Vendelbo MH, Hjorthaug K, Gormsen LC, Munk OL. Clinical feasibility and impact of data-driven respiratory motion compensation studied in 200 whole-body 18f-fdg pet/ct scans. EJNMMI Res. 2022;12(1):16. 35347465 10.1186/s13550-022-00887-x PMC8960547

[6] Büther F, Vehren T, Schäfers KP, Schäfers M. Impact of data-driven respiratory gating in clinical pet. Radiology. 2016;281(1):229–38. 27092660 10.1148/radiol.2016152067

[7] Dawood M, Buether F, Schafers M, Schober O, Schafers K. How many gates? The effect of the number of gates on respiratory gated PET data. J Nucl Med. 2009;50:1471.19690038

[8] Tsutsui Y, Kidera D, Taniguchi T, Akamatsu G, Komiya I, Umezu Y, et al. Accuracy of amplitude-based respiratory gating for pet/ct in irregular respirations. Ann Nucl Med. 2014;28:770–9. 24950753 10.1007/s12149-014-0870-5

[9] Khamis H, Wollenweber S. Motionfree: device-less digital respiratory gating technique, seamlessly integrated in pet imaging routine. GE Healthcare white Paper; 2019. pp. 1–12.

[10] Messerli M, Liberini V, Grünig H, Maurer A, Skawran S, Lohaus N, et al. Clinical evaluation of data-driven respiratory gating for pet/ct in an oncological cohort of 149 patients: impact on image quality and patient management. Br J Radiol. 2021;94(1126):20201350. 34520673 10.1259/bjr.20201350 PMC9328056

[11] Walker MD, Morgan AJ, Bradley KM, McGowan DR. Evaluation of data-driven respiratory gating waveforms for clinical pet imaging. EJNMMI Res. 2019;9:1–10. 30607651 10.1186/s13550-018-0470-9 PMC6318161

[12] Teoh EJ, McGowan DR, Macpherson RE, Bradley KM, Gleeson FV. Phantom and clinical evaluation of the bayesian penalized likelihood reconstruction algorithm q. clear on an lyso pet/ct system. J Nucl Med. 2015;56(9):1447–52. 26159585 10.2967/jnumed.115.159301 PMC4558942

[13] Lohaus N, Enderlin F, Skawran S, Maurer A, Abukwaik AM, Franzen D, et al. Impact of Bayesian penalized likelihood reconstruction on quantitative and qualitative aspects for pulmonary nodule detection in digital 2-[18f] fdg-pet/ct. Sci Rep. 2022;12(1):8308. 35585129 10.1038/s41598-022-09904-4 PMC9117286

[14] Sullivan GM, Artino AR Jr. Analyzing and interpreting data from likert-type scales. J Grad Med Educ. 2013;5(4):541. 24454995 10.4300/JGME-5-4-18 PMC3886444

[15] Napoli NJ, Rodrigues VR, Davenport PW. Characterizing and modeling breathing dynamics: flow rate, rhythm, period, and frequency. Front Physiol. 2022;12:772295. 35264974 10.3389/fphys.2021.772295 PMC8899297

[16] Noto T, Zhou G, Schuele S, Templer J, Zelano C. Automated analysis of breathing waveforms using breathmetrics: a respiratory signal processing toolbox. Chem Senses. 2018;43(8):583–97. 29985980 10.1093/chemse/bjy045 PMC6150778

[17] Werner R, Szkitsak J, Madesta F, Büttgen L, Wimmert L, Sentker T, et al. Clinical application of breathing-adapted 4d ct: image quality comparison to conventional 4d ct. Strahlenther Onkol. 2023;199(7):686–91. 37000223 10.1007/s00066-023-02062-0 PMC10281893

[18] Morley NC, McGowan DR, Gleeson FV, Bradley KM. Software respiratory gating of positron emission tomography-computed tomography improves pulmonary nodule detection. Am J Respir Crit Care Med. 2017;195(2):261–2. 27755923 10.1164/rccm.201607-1371IM PMC5394788

[19] Kang SY, Moon BS, Kim HO, Yoon H-J, Kim BS. The impact of data-driven respiratory gating in clinical f-18 fdg pet/ct: comparison of free breathing and deep-expiration breath-hold ct protocol. Ann Nucl Med. 2021;35(3):328–37. 33449303 10.1007/s12149-020-01574-4

[20] Sun P, Thomas MA, Luo D, Pan T. New full-counts phase-matched data-driven gated (ddg) pet/ct. Med Phys. 2024;51(7):4646–54. 38648671 10.1002/mp.17097 PMC11233242

[21] Wilson Z, Kuhar M, Su K-H, Johnsen R, Bradley KM, McGowan DR. Improved attenuation correction registration for fdg pet/ct images using data-driven gating (ddg)-based motion match. EJNMMI Physics. 2026. Short communication, in revisions. 10.1186/s40658-026-00896-y PMC13433694 42177740

