# Supplementary material for: Deep learning-enhanced data-driven gating improves FDG PET/CT clinical image quality
Source: EJNMMI Phys. 2026 Apr 5;13:46. doi: 10.1186/s40658-026-00851-x (PMC13176395; doi:10.1186/s40658-026-00851-x)
Supplement: Supplementary file 1 — (pdf 123 KB) [file 40658_2026_851_MOESM1_ESM.pdf]

Supplementary Material

Patient Information

Below is a table of the 39 patients included in this study.

Table 10 Patient characteristics

Height (m)	Weight (kg)	Total dose (MBq)
1.91	120	478
1.67	63	255
1.72	84	324
1.56	45	179
1.80	68	284
1.60	42	144
1.71	75	293
1.74	82	329
1.68	70	273
1.59	55	210
1.73	78	306
1.65	60	241
1.77	85	340
1.69	72	282
1.58	50	199
1.70	76	298
1.75	80	315
1.62	58	228
1.79	88	352
1.66	65	260
1.72	74	289
1.68	69	271
1.74	83	331
1.63	57	225
1.71	77	302
1.69	71	276
1.76	86	346
1.64	59	234
1.78	90	361
1.67	66	262
1.73	79	310
1.70	73	285
1.75	81	321
1.61	54	206
1.77	87	349
1.68	68	269
1.74	84	333
1.65	62	248
1.72	76	295
